# Distinctive clinical traits of lupus-related myocarditis: a multicentre retrospective study

**DOI:** 10.1093/rheumatology/keae376

**Published:** 2024-07-24

**Authors:** Giuseppe A Ramirez, Noora E A Holopainen, Maria Gerosa, Giacomo De Luca, Chiara Bellocchi, Daniel Arroyo-Sánchez, Simone Sala, Giovanni Peretto, Luca Moroni, Francesca Mastropaolo, Lorenza M Argolini, Giuseppe Pizzetti, Anna Palmisano, Antonio Esposito, Adriana Cariddi, Silvia Sartorelli, Corrado Campochiaro, Lorenzo Beretta, Enrica P Bozzolo, Roberto Caporali, Lorenzo Dagna

**Affiliations:** Università Vita-Salute San Raffaele, Milan, Italy; Unit of Immunology, Rheumatology, Allergy and Rare Diseases, IRCCS Ospedale San Raffaele, Milan, Italy; Università Vita-Salute San Raffaele, Milan, Italy; Unit of Immunology, Rheumatology, Allergy and Rare Diseases, IRCCS Ospedale San Raffaele, Milan, Italy; Department of Clinical Science of Community Health and Research Center for Adult and Pediatric Rheumatic Diseases, Università degli Studi di Milano, Milan, Italy; Unit of Clinical Rheumatology, ASST Gaetano Pini-CTO, Milan, Italy; Università Vita-Salute San Raffaele, Milan, Italy; Unit of Immunology, Rheumatology, Allergy and Rare Diseases, IRCCS Ospedale San Raffaele, Milan, Italy; Department of Clinical Science of Community Health and Research Center for Adult and Pediatric Rheumatic Diseases, Università degli Studi di Milano, Milan, Italy; Referral Center for Systemic Autoimmune Diseases, Fondazione IRCCS Ca’ Granda Policlinico, Milan, Italy; Unit of Immunology, Rheumatology, Allergy and Rare Diseases, IRCCS Ospedale San Raffaele, Milan, Italy; Department of Immunology, Hospital Universitario 12 de Octubre, Madrid, Spain; Instituto de Investigación Biomédica Hospital 12 de Octubre, Madrid, Spain; Unit of Cardiology, IRCCS Ospedale San Raffaele, Milan, Italy; Unit of Cardiology, IRCCS Ospedale San Raffaele, Milan, Italy; Università Vita-Salute San Raffaele, Milan, Italy; Unit of Immunology, Rheumatology, Allergy and Rare Diseases, IRCCS Ospedale San Raffaele, Milan, Italy; Università Vita-Salute San Raffaele, Milan, Italy; Unit of Immunology, Rheumatology, Allergy and Rare Diseases, IRCCS Ospedale San Raffaele, Milan, Italy; Unit of Clinical Rheumatology, ASST Gaetano Pini-CTO, Milan, Italy; Unit of Cardiology, IRCCS Ospedale San Raffaele, Milan, Italy; Unit of Radiology, IRCCS Ospedale San Raffaele, Milan, Italy; Unit of Radiology, IRCCS Ospedale San Raffaele, Milan, Italy; Unit of Immunology, Rheumatology, Allergy and Rare Diseases, IRCCS Ospedale San Raffaele, Milan, Italy; Unit of Immunology, Rheumatology, Allergy and Rare Diseases, IRCCS Ospedale San Raffaele, Milan, Italy; Università Vita-Salute San Raffaele, Milan, Italy; Unit of Immunology, Rheumatology, Allergy and Rare Diseases, IRCCS Ospedale San Raffaele, Milan, Italy; Department of Clinical Science of Community Health and Research Center for Adult and Pediatric Rheumatic Diseases, Università degli Studi di Milano, Milan, Italy; Referral Center for Systemic Autoimmune Diseases, Fondazione IRCCS Ca’ Granda Policlinico, Milan, Italy; Unit of Immunology, Rheumatology, Allergy and Rare Diseases, IRCCS Ospedale San Raffaele, Milan, Italy; Department of Clinical Science of Community Health and Research Center for Adult and Pediatric Rheumatic Diseases, Università degli Studi di Milano, Milan, Italy; Unit of Clinical Rheumatology, ASST Gaetano Pini-CTO, Milan, Italy; Università Vita-Salute San Raffaele, Milan, Italy; Unit of Immunology, Rheumatology, Allergy and Rare Diseases, IRCCS Ospedale San Raffaele, Milan, Italy

**Keywords:** systemic lupus erythematosus, myocarditis, antiphospholipid antibodies, damage, long-term outcomes

## Abstract

**Objectives:**

Cardiovascular involvement in systemic lupus erythematosus (SLE) is frequent, but little is known about possible distinctive traits of SLE-related myocarditis (myoSLE) in comparison with patients with SLE (onlySLE) or myocarditis alone (onlyMyo).

**Methods:**

A retrospective analysis was performed comparing patients with myoSLE (*n* = 25) from three centres with consecutive patients with onlySLE (*n* = 279) and onlyMyo (*n* = 88). SLE patients were dichotomized by disease duration ≤1 *vs* >1 year into recent onlySLE/early myoSLE *vs* longstanding onlySLE/late myoSLE. Further stratification into disease duration of 1–5, 5–10 and >10 years was also performed. SLE disease activity index 2000 (SLEDAI-2K) was used to estimate disease activity. Myocarditis was diagnosed through biopsy or MRI.

**Results:**

Women were significantly more frequent among myoSLE than among onlyMyo (72% *vs* 43%; *P* = 0.013). Compared with onlyMyo, myoSLE patients had a higher frequency of conduction abnormalities (22% *vs* 5%; *P* *=* 0.046) and presented with numerically higher frequencies of left ventricular function compromise (48% *vs* 30%), along with higher pro-brain natriuretic peptide levels. Inflammation markers were higher in myoSLE compared with onlyMyo and with patients with onlySLE with >10 years of disease duration. SLEDAI-2K was significantly higher in late myoSLE than in longstanding onlySLE. Antiphospholipid syndrome was more frequent in myoSLE than in onlySLE. Multivariate analysis showed an association among myoSLE, anti-β-2-glycoprotein I antibodies (aB2GPI, *P = *0.014) and a higher number of involved British Isles Lupus Assessment Group domains in patient history (*P* *=* 0.003).

**Conclusion:**

myoSLE has unique clinical traits compared with other forms of myocarditis and is associated with aB2GPI and a more severe SLE course.

Rheumatology key messages

- Anti-β-2-glycoprotein I antibodies and involvement of a higher number of BILAG domains in patient history are risk factors for myocarditis in SLE.
- Systemic inflammation is more prominent in myocarditis and SLE than in SLE or myocarditis alone.
- SLE-related myocarditis presents with more significant cardiac dysfunction than other forms of myocarditis.

## Introduction

Systemic lupus erythematosus (SLE) is a multiorgan autoimmune disease characterized by protean clinical manifestations [1]. While loss of tolerance towards cell nuclear components constitutes a pathogenic hallmark of the disease, multiple distinct pathophysiological phenomena may occur among individuals and disease phases, possibly in association with specific genetic factors and serological profiles. Within this framework, SLE clinical presentation usually incorporates otherwise standalone disorders such as cutaneous lupus or autoimmune hepatitis into a more complex syndrome [2]. Conventional diagnostic and therapeutic strategies usually lump this complexity into a homogeneous nosological abstraction or assume that translating knowledge on isolated aspects of SLE clinical spectrum from other clinical contexts might suffice to adapt to SLE pathophysiology. Unfortunately, the limitations of this simplistic approach are clearly highlighted by the low success rate of pharmacological trials in SLE and the modest value of disease non-specific clinimetrics in estimating the risk of SLE-related complications [3–5].

Myocarditis constitutes an additional example of an immune-mediated organ- and potentially life-threatening condition, which can be sustained by microbial or pharmacological triggers [6] but also occur idiopathically either as an isolated manifestation or within the broader context of systemic diseases, including SLE [7]. Myocarditis usually presents with chest pain, dyspnoea and palpitations, although subclinical courses may also be observed. In this cases, cardiac magnetic resonance imaging (MRI) has been shown to provide incremental prognostic value compared with heart ultrasound [8]. Myocarditis prognosis is usually related to the degree of left ventricular ejection fraction impairment and severity of potential arrhythmia [9]. Comorbid systemic immune-mediated disorders are associated with poorer myocarditis outcomes [10, 11]. Amid this broader set of pathological conditions, myocarditis arising in the setting of eosinophilic syndromes and histiocytoses have more clearly been defined from a pathophysiological and management standpoint, thanks to the unique characteristics of their inflammatory infiltrate [12–15]. Less is known about potential distinctive features of myocarditis developing in the context of connective tissue diseases, although histological studies indicate that disease-specific pathophysiological traits may affect the shape of myocardial inflammation [15, 16].

Cardiovascular morbidity constitutes a frequent, yet incompletely understood, feature of SLE clinical spectrum and a major cause of disability and reduced survival. Recent estimates suggest that the prevalence of ultrasonographic heart abnormalities exceeds 40% in patients with SLE, which is comparable to the prevalence of lupus nephritis [17]. Myocardial inflammation is reported to affect 1–10% of patients with SLE at a clinical level [18, 19], with a higher frequency of pathological signs potentially identifiable in post-mortem studies [7]. Lupus myocarditis can cause permanent impairment in heart contractile function, leading to chronic disability. In addition, mortality associated to lupus myocarditis is reportedly high [18, 20, 21]. Nonetheless, limited evidence exists about potential distinctive traits of patients with SLE-related myocarditis (myoSLE) in contrast to patients with SLE without myocarditis (onlySLE) and patients with non-SLE autoimmune myocarditis (onlyMyo) [16]. Accordingly, myoSLE shares the problem of attribution to the parent systemic disease with other non-specific, yet typical, SLE manifestations, such as neuropsychiatric events [22]. To address this issue and gather potentially novel information on the specificity of myoSLE we set up a multicentre comparative study based on retrospective clinical chart review.

## Methods

### Patients and clinical variables

This study involved patients with SLE according to the 2012 SLE International Collaborating Clinics (SLICC) criteria [23] and/or with myocarditis, defined as biopsy proven evidence of myocardial inflammation or MRI signs consistent with active or past myocarditis as per the revised Lake Louise criteria [24]. Subjects with myoSLE were identified by chart review among patients with SLE followed up in the SMiLE (Milan Lupus Consortium) Centres (IRCCS Ospedale San Raffaele, ASST Pini CTO and Policlinico di Milano). Patients were enrolled upon written informed consent in a multicentre observational protocol (‘MLC’) conforming to the Declaration of Helsinki and approved by San Raffaele Institutional Review Board (reference 84/INT/2019) and Comitato Etico Milano Area 2 (reference number 0002450/2020). These patients were compared with a cohort of consecutive patients with onlySLE and a cohort of consecutive patients with onlyMyo being followed up at IRCCS Ospedale San Raffaele and enrolled upon informed consent in a broader observational protocol, which conforms to the Declaration of Helsinki, approved by San Raffaele Institutional Review Board (PanImmuno Research Protocol, reference number 22/INT/2018) and aimed at comparing disease characteristics among immune-mediated diseases.

Clinical data collection included demographics, general disease characteristics and treatment history up to the last visit (onlySLE) or myocarditis onset (onlyMyo, myoSLE). Patients with SLE were also dichotomized into patients with disease duration within (recent onlySLE, early myoSLE) *vs* exceeding 1 year (longstanding onlySLE, late myoSLE) at time of last assessment. Further stratification into disease duration of 1–5, 5–10 and >10 years was also performed. Conventional cardiovascular risk factor (smoking, dyslipidaemia, hypertension, diabetes) prevalence was also collected, along with history of thromboembolic/ischaemic cardiac or non-cardiac events. Prevalence of cardiovascular risk factors and comorbidities were compared among groups.

Myocarditis-specific variables included symptoms/signs at presentation, pro-brain natriuretic peptide (proBNP), troponin T and C-reactive protein values at myocarditis onset, topographical information on the extent of myocardial inflammation by ultrasound and/or MRI, specific MRI and histopathological features, evidence of Holter ECG abnormalities. MRI variables included estimation of cardiac chamber volumes and function, presence of oedema through T2-weighted Short-tau Inversion Recovery (STIR) sequences, and early and late gadolinium enhancement (EGE and LGE, respectively). LGE was further categorized into diffuse *vs* patchy LGE, subepicardial *vs* subendocardial LGE and LGE involving the right *vs* the left ventricle. Histopathological features encompassed active *vs* chronic inflammation, evidence of oedema, necrosis or fibrosis, signs of thrombosis or vascular inflammation. Due to high variability among laboratories in terms of troponin T and proBNP levels, both variables were only analysed dichotomously, that is by considering values below *vs* above the upper level of normality. SLE-specific features encompassed evidence of anti-cardiolipin (aCL), anti-β-2-glycoprotein I (aB2GPI), anti-DNA (ADNA), low complement and/or lupus anticoagulant (LAC). Patients were classified by their antiphospholipid antibody (aPL) profile and history of aPL-syndrome (APS) according to the Sydney criteria [25]. Disease activity was estimated by the SLE disease activity index 2000 (SLEDAI-2K) [26] and damage with the SLICC/American College of Rheumatology (ACR) damage index (SDI) [27]. Disease extent was calculated as the number of British Isles Lupus Assessment Group (BILAG) 2004 domains having ever been involved in patient history at time of observation. Clinimetrics were calculated and digitalized through in-house software (Clinimatrix©), developed in Microsoft Excel [5]. Data regarding the anti-Ro (aSSA), anti-La (aSSB), anti-Smith (aSm), anti-Scl70, anti-Jo1 and anti-ribonucleoprotein (aRNP) antibody profile were collected from all three groups.

### Statistical analysis

The Shapiro–Wilk test was used to test continuous variables for normality. Trends of non-normally distributed continuous variables among groups were assessed by the Mann–Whitney or Kruskal-Wallis test in case of two or more groups. Student’s *t*-test or ANOVA was employed for the same purposes in case of normally distributed variables. χ^2^ test was used to test the association of categorical variables with the groups of interest. Fisher’s exact correction was applied as appropriate. Correlation analyses were performed with Spearman’s test. STATA 15.1 (StataCorp, College Station, TX, USA), Microsoft Excel 2019 and JASP 0.14 were used for the analyses. Data are expressed as percentages or median (interquartile range), unless otherwise specified.

## Results

### General clinical characteristics of patients with myoSLE

By screening 637 patients with SLE, we identified 25 confirmed cases of myoSLE (point prevalence = 3.9%). Eighteen patients with myoSLE were women. In patients with myoSLE, myocarditis onset occurred after 1 (0–10) years from SLE diagnosis. Specifically, 13 patients had myocarditis within 1 year from SLE onset (early myoSLE). Of the remaining 12 patients with late myoSLE, three developed myocarditis within 1–5 years, three within 5–10 years and six after >10 years from SLE diagnosis. Most cases presented with chest pain (12/25) and/or dyspnoea (12/25), while 5/25 patients had a subclinical course (details on their clinical history are provided in Supplementary Table S1, available at *Rheumatology* online). Three patients presented with arrhythmia and 6/18 undergoing Holter ECG had pathological findings, including 3/6 rhythm anomalies and 4/6 conduction anomalies. One patient had an episode of cardiac arrest during sepsis 1 year after the onset of myocarditis. She was successfully resuscitated. There was no imaging evidence of relapsing active myocarditis at that time. Another patient died due to refractory asystolia possibly due to myocarditis one year after onset. In 2/25 cases (8%), myocarditis occurred concomitantly with heart ischaemia and 14/25 had concomitant pericarditis. In terms of myocarditis extent, 8/25 had diffuse heart involvement while 17/25 had localized inflammation. By MRI or ultrasound imaging, the inferior region of the heart was the most frequently involved site (14/25), followed by the interventricular septum and lateral region (both 11/25). Functionally, almost half of the patients (12/25) presented with left ventricular ejection fraction below 55%. The median SLEDAI-2K at myocarditis onset was 8 (4–14), while the median SDI was 0 (0–1).

Most patients were treated with beta-blockers (10/25), six received angiotensin converting enzyme inhibitors, one ivabradine and one clonidine. All patients received high-dose corticosteroids. Most patients (9/25) were treated with mycophenolate mofetil, three received azathioprine, two intravenous cyclophosphamide and one subcutaneous methotrexate.

Patients with myoSLE were compared with 279 consecutive subjects with onlySLE and 88 consecutive subjects with onlyMyo. Among these subjects, 69/88 had no other immune-mediated comorbidity, while 19 had one or more immune-mediated disorders (Supplementary Table S2, available at *Rheumatology* online). Patients with SLE with and without myocarditis had similar demographics, while patients with myoSLE were more frequently women compared with onlyMyo (72% *vs* 43%; *P* *=* 0.013). The age of SLE onset and of myocarditis onset did not show significant differences when myoSLE were compared with onlySLE and onlyMyo patients, respectively. Conventional cardiovascular risk factor prevalence was also similar among groups (Table 1).

**Table 1.** Demographics and conventional cardiovascular risk factors

	myoSLE (*n* = 25)	onlySLE (*n* = 279)	onlyMyo (*n* = 88)
Demographics			
Women, *n* (%)	18 (72)*	226 (81)	38 (43)
Age at SLE onset, median (IQR), years	26 (22–33)	29 (21–38)	NA
SLE duration, median (IQR), years	1 (0–10)**	10 (3–20)	NA
≤1 year	12 (48)**	19 (7)	NA
>1 year	13 (52)**	261 (93)	NA
≤5 years	3 (12)	63 (23)	NA
≤10 years	3 (12)	49 (18)	NA
>10 years	6 (24)	144 (51)	NA
Age at myocarditis onset, median (IQR), years	37 (24–45)	NA	45 (36–56)
Cardiovascular risk factors			
Smoker (ever),*n* (%)	10 (40)	97 (35)	44 (50)
Hypertension, *n* (%)	3 (12)	39 (14)	21 (24)
Dyslipidaemia, *n* (%)	8 (32)	96 (34)	23 (26)
Diabetes, *n* (%)	0 0	11 (4)	5 (6)

* *P* < 0.05 compared with onlyMyo;

** *P* < 0.001 compared with onlySLE. IQR: interquartile range; myoSLE: SLE-related myocarditis; onlyMyo: myocarditis alone; onlySLE: SLE alone.

### Distinctive traits of myoSLE *vs* onlyMyo

There were no significant differences among myoSLE and onlyMyo in terms of myocarditis extent and clinical presentation. Sixteen patients with myoSLE and six patients with onlyMyo had detectable anti-extractable nuclear antigen antibodies (aENA). Imaging features at cardiac MRI did not show significant differences when patients with myoSLE and onlyMyo were compared, although a patchy LGE pattern was numerically more frequent in patients with onlyMyo (41%) than in patients with myoSLE (16%; *P* *=* 0.056; Table 2). Rhythm abnormalities were more frequently reported in patients with onlyMyo (37/73) than in patients with myoSLE (3/18; *P* *=* 0.015). Conversely, conduction abnormalities were more prevalent among patients with myoSLE than patients with onlyMyo (4/18 *vs* 4/73; *P* *=* 0.046). Histological information was available for 5/25 patients with myoSLE and 70/88 with onlyMyo. Exploratory comparative analyses between the two groups showed an equal representation of acute features, hallmarks of inflammation and fibrosis in both groups, while tissue oedema was less frequent in myoSLE (1/5) than in onlyMyo (50/70; *P* *=* 0.034). None of the five available myocardial biopsies from patients with myoSLE showed evidence of necrosis, compared with 44/70 from patients with onlyMyo (*P* *=* 0.010). Thrombotic features were detected in 2/70 patients with onlyMyo and 0/5 patients with myoSLE. Conversely, a history of cardiac or extra-cardiac thrombotic events was significantly more frequent in patients with myoSLE (10/25) than patients with onlyMyo (5/88; *P* < 0.001; Table 2).

**Table 2.** Myocarditis features

	myoSLE (*n* = 25)	onlyMyo (*n* = 88)
Clinical features, *n* (%)		
Subclinical myocarditis	5 (20)	30 (34)
Chest pain	12 (48)	37 (42)
Palpitations	10 (40)	23 (26)
Dyspnoea	12 (48)	34 (39)
Arrhythmia	3 (12)	26 (30)
Cardiac arrest	1 (4)	3 (3)
Concomitant pericarditis	14 (56)	33 (38)
Concomitant cardiac ischaemia	2 (8)	7 (8)
Death due to myocarditis, *n* (%)	1 (4)	0 0
Myocarditis extent, *n* (%)		
Apex	7 (28)	24 (27)
Lateral region	11 (44)	50 (57)
Septal region	11 (44)	41 (47)
Anterior	5 (20)	15 (17)
Posterior	7 (28)	16 (18)
Inferior	14 (56)	49 (56)
Left ventricular ejection fraction (%), median (IQR)	49 (30–60)	56 (50–61)
Left ventricular ejection fraction <55%, *n* (%)	12 (48)	26 (30)
Holter ECG abnormalities, *n* (%)	6 (24)	39 (44)
Rhythm abnormalities	3 (12)*	37 (42)
Conduction abnormalities	4 (16)*	4 (5)
MRI features, *n* (%)		
Oedema at any site	13 (52)	47 (53)
Early Gd enhancement	2 (8)	3 (3)
Late Gd enhancement	15 (60)	64 (73)
Patchy LGE	4 (16)	36 (41)
Subepicardial LGE	9 (36)	44 (50)
Subendocardial LGE	1 (4)	7 (8)
Right ventricle LGE	1 (4)	0 0
Biopsy features, *n* (%)		
Active inflammation	3 (60)	48 (63)
Chronic inflammation	3 (60)	38 (50)
Necrosis	0 0*	44 (58)
Thrombosis	0 0	2 (3)
Fibrosis	2 (40)	55 (72)
Vasculitis	0 0	0 0
Oedema	1 (20)*	50 (66)
Laboratory features		
Abnormal proBNP, *n* (%)	12 (86)**	29 (38)
Abnormal troponin T, *n* (%)	15 (88)	49 (64)
ESR, median (IQR), mm/h	55 (45–87)***	8 (3–18)
CRP, median (IQR), mg/l	14 (7–41)***	3 (1–9)

* *P* < 0.05,

** *P* < 0.010,

*** *P* < 0.001. IQR: interquartile range; LGE: late gadolinium enhancement; myoSLE: SLE-related myocarditis; onlyMyo: myocarditis alone; proBNP: pro-brain natriuretic peptide.

In patients with myoSLE, altered proBNP levels were found in 12/14 patients with available information, which was higher than the prevalence of proBNP alterations in patients with onlyMyo (29/63; *P* *=* 0.008). Depression of left ventricular ejection fraction below 55% was numerically more frequent in patients with myoSLE (12/25) than in patients with onlyMyo (26/88; *P* *=* 0.141). Alterations in troponin T levels did not show differences between the two groups. Patients with myoSLE had significantly higher ESR and CRP levels at presentation than patients with onlyMyo (*P* < 0.001 for both variables; Table 2).

Patients with myoSLE were more frequently treated with mycophenolate mofetil than patients with onlyMyo (9/25 *vs* 8/88; *P* *=* 0.005), while azathioprine (3/25 *vs* 50/88; *P* < 0.001) and beta-blockers (10/25 *vs* 62/88; *P* *=* 0.012) were less frequently employed. There were no significant differences in terms of use of ivabradine and amiodarone among patients with myoSLE and onlyMyo.

### Distinctive traits of myoSLE *vs* onlySLE

**Figure 1.**
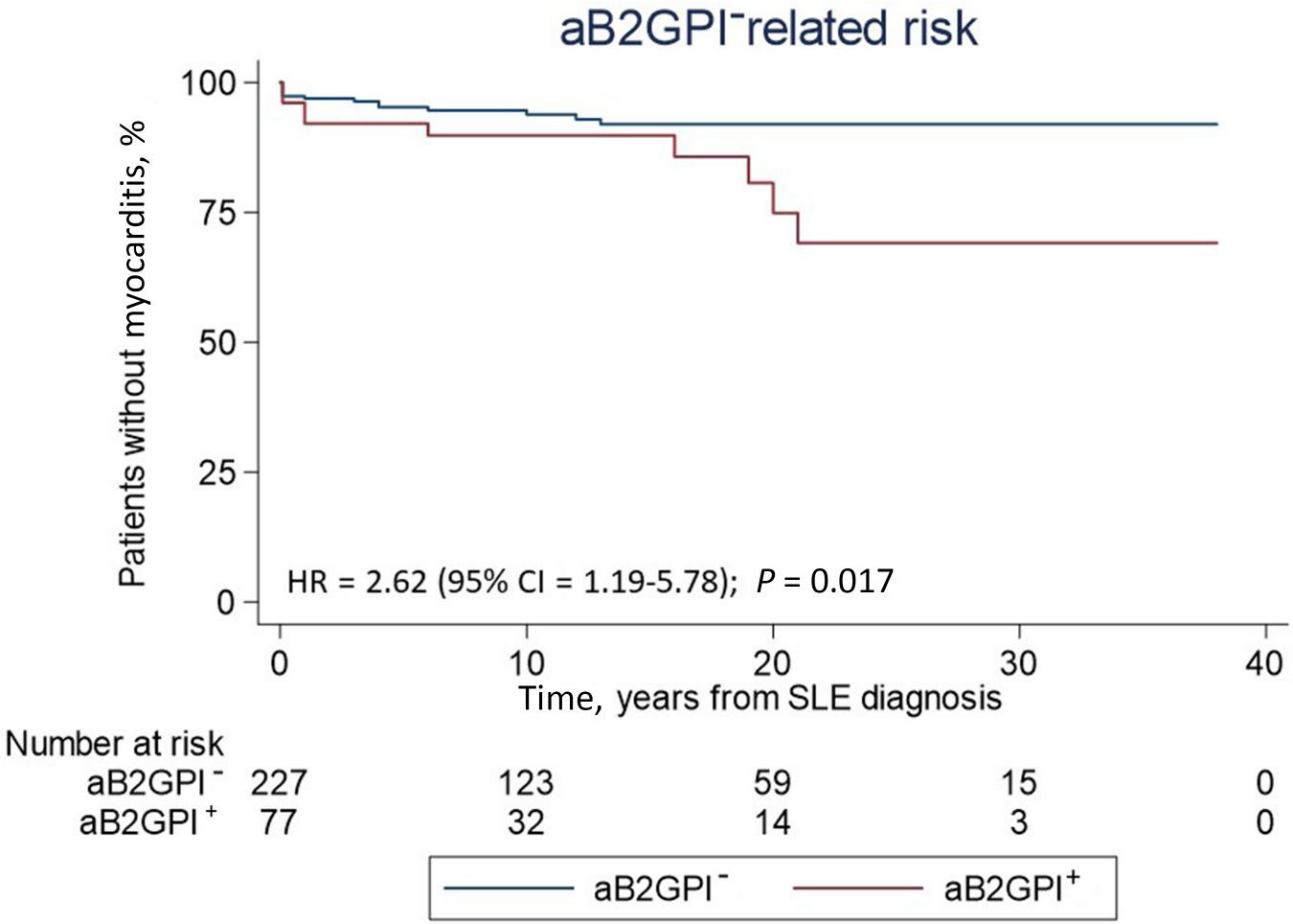
Association between aB2GPI and development of myocarditis in SLE. Kaplan–Meyer survival curves showing the risk of developing myocarditis in patients with SLE stratified by their anti-β-2-glycoprotein I (aB2GPI) profile. In this analysis, patients with myoSLE were pooled with patients with onlySLE. Patients with SLE were dichotomized into patients with positive vs negative aB2GPI (aB2GPI^+^, aB2GPI^−^, respectively) and myocarditis was set as the failure event. HR: hazard ratio

Patients with myoSLE had a higher number of involved disease domains in their clinical history than patients with onlySLE. Beside the cardiopulmonary domain, mucocutaneous and gastrointestinal manifestations were more frequent in myoSLE than in onlySLE. More than one-third of patients with myoSLE had a history of APS, compared with 10% in patients with onlySLE (χ^2^ = 14.470; *P* *=* 0.001). Accordingly, aB2GPI were significantly more frequent in myoSLE compared with onlySLE (Fig. 1), with similar trends observed for other aPL. There were no differences in treatment history among patients with myoSLE and onlySLE (Table 3). By multivariate Cox’s regression analysis, both disease extent by number of involved BILAG domains (HR = 1.46; 95% CI: 1.14, 1.86; *P* *=* 0.003) and an aB2GPI positive profile (HR = 2.31; 95% CI: 1.04, 5.14; *P* *=* 0.014) were independently and significantly associated with myocarditis onset among patients with SLE.

**Table 3.** SLE features

	myoSLE (*n* = 25)	onlySLE (*n* = 279)
Clinical domain prevalence, *n* (%)		
Musculoskeletal	21 (84)	213 (76)
Mucocutaneous	22 (88)^*^	183 (66)
Renal	12 (48)	102 (37)
Haematological	14 (56)	178 (64)
Neuropsychiatric	8 (32)	44 (16)
Cardiopulmonary	25 (100)^***^	89 (32)
Constitutional	18 (72)	201 (72)
Gastrointestinal	5 (20)^*^	17 (6)
Ophthalmic	1 (4)	10 (4)
Number of involved BILAG domains, median (IQR)	5 (4–6)^***^	4 (3–5)
Antiphospholipid syndrome, *n* (%)	9 (36)^**^	28 (10)
Serological features, *n* (%)		
aPL	14 (56)	112 (40)
aCL	11 (44)	100 (36)
aB2GPI	11 (44)^*^	66 (24)
LAC	8 (32)	73 (26)
adsDNA	18 (72)	222 (80)
aSSA	8 (32)	104 (37)
aSSB	1 (4)	46 (17)
aRNP	5 (20)	56 (21)
aScl70	1 (4)	5 (3)
aJo1	0 (0)	0 (0)
Treatment history, *n* (%)		
HCQ	24 (96)	260 (93)
MTX	3 (12)	64 (23)
MMF	15 (60)	115 (41)
AZA	10 (40)	142 (51)
CYA	3 (12)	25 (9)
CYC	8 (32)	67 (24)
BEL	2 (8)	56 (20)
RTX	1 (4)	18 (6)

aB2GPI: anti-β-2-glycoprotein I; aCL: anti-cardiolipin; adsDNA: anti-double stranded DNA; aPL: antiphospholipid antibody; aSSA: anti-Ro; aSSB: anti-La; aRNP: anti-ribonucleoprotein; BEL: belimumab; BILAG: British Isles Lupus Assessment Group; CYA: cyclosporine A; IQR: interquartile range; LAC: lupus anticoagulant; myoSLE: SLE-related myocarditis; onlyMyo: myocarditis alone; RTX: rituximab.

**Figure 2.**
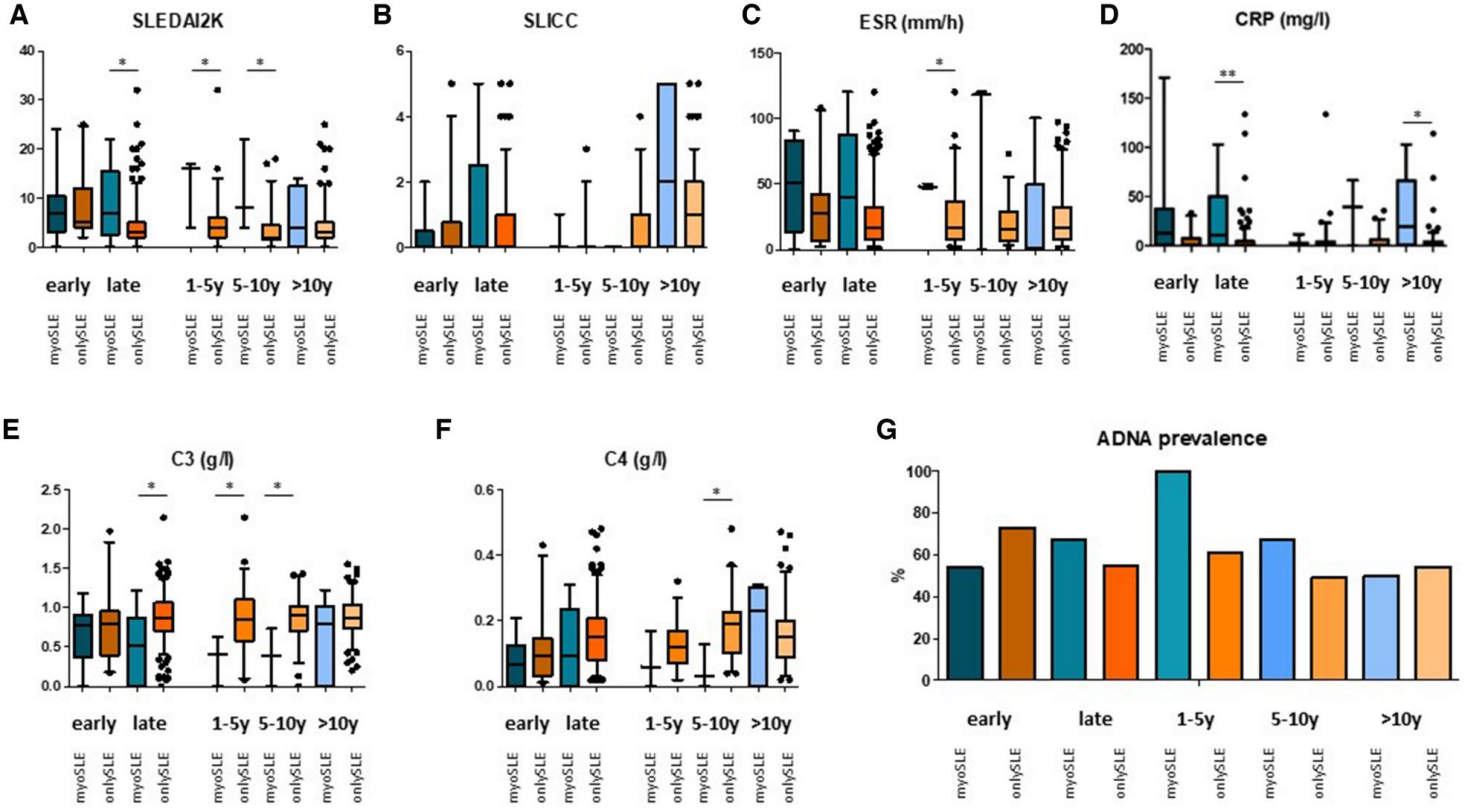
SLE features by disease duration groups. In this figure, patients with myoSLE (green–blue fade, first elements of each couple of data) and onlySLE (orange–light orange fade, second elements of each couple of data) are stratified by disease duration. (**A–F**) Boxplots showing the distribution of quantitative variables among groups. These variables include the SLE disease activity index 2000 version (SLEDAI-2K), the SLE International Collaborating Clinics/American College of Rheumatology damage index (SDI) along with ESR, CRP, complement C3 and C4 levels at time of assessment. (**G**) Bar chart showing the prevalence of positive ADNA at time of assessment. Patients with early manifestations (first couples of boxes and whiskers or bars) include patients with SLE duration within 1 year. The parameters of patients with late manifestations (late myoSLE or longstanding SLE) are represented by the second couples of boxes and whiskers or bars and are further subdivided into three classes corresponding to the following couples of boxes and whiskers. *P < 0.05, **P < 0.01. ADNA: anti-DNA; myoSLE: SLE-related myocarditis; onlyMyo: myocarditis alone

When patients with SLE (onlySLE and myoSLE) were stratified by disease duration ranges, numerically higher SLEDAI-2K and SDI levels along with lower complement levels and higher inflammation markers were observed in patients with myoSLE at all time points. SLEDAI-2K was significantly higher in patients with late myoSLE compared with longstanding onlySLE. Consistent with this, significantly lower C3 levels were observed in the same subgroups. CRP levels were significantly higher in patients who had myocarditis after >10 years of SLE than patients without myocarditis and similar disease duration. Detectable ADNA were equally prevalent among groups (Fig. 2). The aENA profile did not differ between myoSLE and onlySLE (Table 3).

## Discussion

In this study, we found that patients with myoSLE were demographically more similar to patients with onlySLE than to patients with onlyMyo, while showing a significantly higher prevalence of aB2GPI and a broader disease extent compared with patients with onlySLE. Patients with myoSLE were also found to present with higher proBNP levels, despite similar myocarditis extent, compared with onlyMyo and with higher levels of systemic inflammation markers compared with both groups.

Previous comparative analyses within SLE cohorts suggest that patients developing myocarditis have a more severe disease profile than onlySLE patients [19, 28]. Consistent with this, we found that a broader disease extent was independently associated with the risk of developing myocarditis over time. Furthermore, we observed that patients with myocarditis had higher disease activity scores and more significant alterations in inflammation markers when compared with patients with SLE of similar disease duration. Specifically, patients with late myoSLE had more active disease compared with longstanding onlySLE. Patients with persisting SLE activity even in later stages of the disease are at increased risk of damage accrual and complication development [29, 30]. Consistent with this, myocarditis has a negative prognostic impact in patients with SLE [19].

Besides observing an association between myoSLE and enhanced SLE severity, we also found that patients with myoSLE presented more frequently with laboratory signs of cardiac overload and possibly with more severe depression of left ventricular ejection fraction, despite similar symptom profiles between the two groups. Conduction abnormalities were also disproportionately higher in patients with myoSLE compared with patients with onlyMyo, who in turn had a higher frequency of rhythm abnormalities. Conduction abnormalities are reported to affect up to two-thirds of patients with SLE [31] and constitute a frequent manifestation of myocarditis in the setting of autoimmune diseases [32]. These findings, along with evidence of higher ESR levels at myocarditis onset in patients with myoSLE, might indicate that SLE-specific immune phenomena might synergize with stereotyped inflammatory events occurring in the context of standalone myocarditis towards a more severe functional phenotype [33]. In addition, conduction abnormalities might have affected treatment choices towards a less frequent use of beta-blockers, which in turn might account for worse functional performance in patients with myoSLE. Limited evidence from histology, showing lower rates of necrosis in myoSLE compared with onlyMyo, might also suggest that these functional alterations can be more easily reverted in patients with SLE, provided that timely and adequate treatment is instated. Indeed, CRP levels were also disproportionately elevated in patients with myoSLE compared with onlySLE and onlyMyo patients. This finding contrasts with the typical low-CRP profile observed in patients with SLE. Aberrant type I interferon responses constitute a pathogenic hallmark of SLE and are usually associated with antiviral-like low-CRP responses [34], possibly suggesting that additional inflammatory pathways are overactivated in patients with SLE who develop myocarditis. The IL-1 pathway is associated with high-CRP responses [35] and has been claimed to play a shared pivotal role across different forms of myocarditis [36, 37], including lupus myocarditis [38]. IL-1 production involves the activation of the inflammasome and aberrant inflammasome activation, especially under pathogen-related stimuli, has been described in patients with SLE [39]. Consistent with this, lupus myocarditis is part of the spectrum of SLE manifestations occurring during pathogen-induced flares [40], in contrast to vaccination [41].

In line with evidence of an association between aPL and cardiac involvement in SLE [17, 42], we found that patients with myoSLE had a disproportionately high frequency of aB2GPI (along with a history of APS) compared with patients with onlySLE. Antiphospholipid antibodies constitute a well-known cause of cardiovascular and thromboembolic morbidity in patients with SLE and APS [43, 44] possibly indicating that alterations of haemostasis might specifically contribute to myocarditis in the setting of SLE and APS [7, 45]. In contrast to this hypothesis, the prevalence of concomitant cardiac ischaemia did not differ between myoSLE and onlyMyo in our study, and none of the few available cardiac biopsies from patients with myoSLE had signs of thrombosis. Indeed, mechanistic studies suggest that aPL in general and aB2GPI in particular not only affect haemostasis, but also interfere with autoantigen clearance [46] and facilitate the formation of neutrophil extracellular traps (NETs), ultimately promoting autoimmunity and inflammation [47]. Clinical data indicate that a positive aPL profile correlates with more extensive organ involvement and higher risk of thrombotic and non-thrombotic manifestations in patients with SLE [42, 48, 49], in line with our findings.

Due to the availability of only a few myocardial biopsy samples, we were unable to perform extensive comparative analyses on the histological features potentially distinguishing myoSLE from onlyMyo, which also limits mechanistic considerations about the pathogenic events specifically occurring in myoSLE. Our work has additional limitations in terms of its design. First, data were analysed retrospectively, which limits the strength of our multivariate model for the identification of risk factors for myocarditis development in SLE. In addition, changing diagnostic sensitivity for myocarditis over time with the development of current criteria might have affected the timing of diagnosis in patients with longstanding SLE. Second, although our myoSLE group is representative of a relatively large basin of patients with SLE [50], its sample size remains small, preventing more refined analyses on less frequent clinical, histological and/or laboratory features. Notwithstanding these limitations, our three-directional approach comparing myoSLE with onlySLE and onlyMyo integrates previous discrete evidence in the literature, possibly paving the way to a more comprehensive understanding of the specificity of myoSLE in the context of SLE and myocarditis. Leveraging on association analyses from patients with myocarditis to develop effective attribution algorithms for clinical and research purposes (such as in the case of neuropsychiatric SLE [22]) constitutes a fascinating prospect for future research.

## Supplementary Material

keae376_Supplementary_Data

## Data Availability

Data supporting this work can be shared upon reasonable requests to the corresponding author.
