# Supplementary material for: Distinctive clinical traits of lupus-related myocarditis: a multicentre retrospective study
Source: Rheumatology (Oxford). 2024 Jul 24;64(4):1904–11. doi: 10.1093/rheumatology/keae376 (PMC11962914; doi:10.1093/rheumatology/keae376)
Supplement: keae376_Supplementary_Data [file keae376_supplementary_data.docx]

**Supplemental Table 1: clinical presentation and diagnostic paths in patients with subclinical myocarditis**

| **Patient** | **Clinical events** |
| --- | --- |
| #2 | Ultrasonographic evidence of reduced cardiac contractility at routine follow up heart ultrasound for a history of anti-phospholipid syndrome with Libman-Sacks’ endocarditis. Further characterisation by cardiac magnetic resonance. |
| #7 | Raised troponin T and proBNP at broad-spectrum screening for organ involvement after disseminatet herpetic infection. Confirmation by cardiac magnetic resonance and myocardial biopsy, which showed signed of active non-viral myocarditis. |
| #8 | Raised troponin T and proBNP at broad-spectrum screening for organ involvement at disease onset. Confirmation by cardiac magnetic resonance. |
| #14 | Raised troponin T levels at screening for cardiac involvement in a patient with a disease flare with myositic manifestations. Confirmation by cardiac magnetic resonance. |
| #16 | Raised troponin T levels at screening for cardiac involvement in a patient with reported loss of strength and lactate dehydrogenase elevation. Confirmation by cardiac magnetic resonance. |

**Supplemental Table 2: immune-mediated comorbidities in patients with onlyMyo**

| **Immune-mediated disease** | **N (%)** |
| --- | --- |
| None | 69 (78) |
| Connective tissue disorders | 11 (13) |
| Inflammatory myopathies | 7 (8) |
| Anti-synthetase syndrome | 4 (5) |
| Polymyositis | 1 (1) |
| Dermatomyositis | 2 (2) |
| Systemic sclerosis | 3 (3) |
| Undifferentiated connective tissue disease | 1 (1) |
| Primary anti-phospholipid syndrome | 1 (1) |
| ANCA-associated vasculitides | 3 (3) |
| Others | 4 (4) |
| Graft vs host disease | 1 (1) |
| Coeliac disease | 1 (1) |
| Sarcoidosis | 2 (2) |
